# Microwave-Assisted Defibrillation of Microalgae

**DOI:** 10.3390/molecules26164972

**Published:** 2021-08-17

**Authors:** Frederik L. Zitzmann, Ewan Ward, Xiangju Meng, Avtar S. Matharu

**Affiliations:** Green Chemistry Centre of Excellence, Department of Chemistry, University of York, York YO10 5DD, UK; flz500@york.ac.uk (F.L.Z.); ew1057@york.ac.uk (E.W.); xm657@york.ac.uk (X.M.)

**Keywords:** microalgae, microwave processing, defibrillated cellulose, zero waste biorefinery

## Abstract

The first production of defibrillated celluloses from microalgal biomass using acid-free, TEMPO-free and bleach-free hydrothermal microwave processing is reported. Two routes were explored: i. direct microwave process of native microalgae (“standard”), and ii. scCO_2_ pre-treatment followed by microwave processing. ScCO_2_ was investigated as it is commonly used to extract lipids and generates considerable quantities of spent algal biomass. Defibrillation was evidenced in both cases to afford cellulosic strands, which progressively decreased in their width and length as the microwave processing temperature increased from 160 °C to 220 °C. Lower temperatures revealed aspect ratios similar to microfibrillated cellulose whilst at the highest temperature (220 °C), a mixture of microfibrillated cellulose and nanocrystals were evidenced. XRD studies showed similar patterns to cellulose I but also some unresolved peaks. The crystallinity index (CrI), determined by XRD, increased with increasing microwave processing temperature. The water holding capacity (WHC) of all materials was approximately 4.5 g H_2_O/g sample. The materials were able to form partially stable hydrogels, but only with those processed above 200 °C and at a concentration of 3 wt% in water. This unique work provides a new set of materials with potential applications in the packaging, food, pharmaceutical and cosmetic industries.

## 1. Introduction

Microalgae are unicellular organisms that can be grown both in open water and enclosed systems (photobioreactors) [1]. Certain microalgae are fast-growing carbon fixers, and when grown in large enough quantities sequester CO_2_ [2]. Microalgal biomass is often rich in lipids, protein content and various bioactive components, such as pigments, flavonoids and (poly)phenolics. For example, there is significant research on scCO_2_ extraction of microalgae for lipid extraction to afford extracts rich in fatty acids; palmitic, palmitoleic, linoleic, linolenic, and eicosapentaenoic acid (EPA), as well as carotenoids and α-tocopherols [3]. The composition of microalgal extractives can be selectively enriched by manipulating the growth and cultivation cycles of the microalgae [4,5].

However, many microalgal species have thick, rigid cell walls, which often prevent or limit the extraction of some components, for example, high molecular-weight proteins [6]. Thus, the implementation of cell disruption methods to disintegrate the cell wall and release the intracellular constituents is often necessary. The cell wall of microalgae is primarily composed of cellulose, hemicelluloses, pectins, glycoproteins, and lipids [7,8,9]. The effect of de-pressurization from supercritical carbon dioxide conditions to standard temperature and pressure on microalgal cells is known to amplify the yield of pigments as the compressed CO_2_ rapidly forces itself out of the cell causing cell lysis [10]. Microwave-assisted extraction (MAE) also induces cell disruption to enhance extraction yields. It effectively disrupts the cell wall by exerting a pressure wave caused by the dielectric heating of water within the cells. At high temperatures microwave treatment induces hydrothermal lysis of polysaccharides, which weakens the cell wall and aids in cell disruption [11,12,13].

The extraction of high value bioactive components is the focus of many academic and industrial studies. However, a major crux is overcoming expensive upstream processing (growth and cultivation) and downstream (extraction and purification) costs. Limited attention has been paid to the post-extraction, residual, cellulosic matter because it is not valued (economically) as highly as certain bioactive molecules. To succeed, microalgal biomass needs to be valorized fully, i.e., a zero-waste biorefinery approach is required. Defibrillated celluloses in the form of micro- and nanocellulose are gaining significant importance because of their interesting functional properties: high colloidal stability, high thermal stability, and high mechanical strength. Thus, these materials are useful in a wide range of applications, for example, coatings, optically transparent materials, aerogels, rheology modifiers, electronics, filters, packaging, or molecular scaffolding [14].

Traditionally nanocellulose is produced via intensive chemical and mechanical processing of high cellulosic content biomass, such as, wood pulp [14,15]. Microwave hydrothermal treatment is considered a fast and less energy intensive method than traditional approaches, enabling the production of defibrillated celluloses without the use of any chemical or biological additives. The removal of hemicellulose, pectins, and amorphous cellulose is induced through microwave energy, resulting in defibrillated cellulose fibres with a high degree of crystallinity. This process has been successfully achieved in a range of biomass types including orange peel [13], spent ginger waste [16], and spent pea biomass [14]. The microwave-assisted hydrolysis of hemicellulose, entangling the cellulose microfibrils, was achieved below 180 °C, whereas beyond 180 °C, the hydrolysis of amorphous cellulose and the dispersion of cellulosic fibres were witnessed [13,14,16].

Considering the structural differences between lignocellulosic biomass and microalgae, the latter contain little to no lignin content. Thus, the production of nanocellulose should be less challenging. However, there are only a few reports in the literature that discuss the formation of nanocellulose from microalgae—but with the use of chemicals and/or biological additives. For example, Lee et al. report the production of nanocellulose from microalgae using 2,2,6,6-tetramethylpiperidine-1-oxyl (TEMPO) as a free-radical chemical reagent [17]. TEMPO is corrosive and toxic to aquatic life and, in line with the 12 principles of green chemistry, the use of auxiliaries, especially those that are toxic, should be minimized or eliminated. 

**Figure 1:**
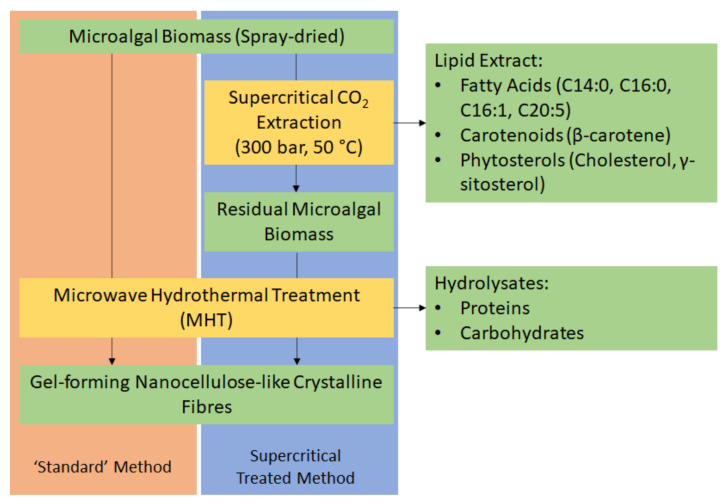
Process flow diagram of a potential biorefinery route of microalgal biomass. NB. ‘Standard’ refers to without any scCO_2_ pretreatment.

Herein, we report the first ever production of defibrillated celluloses from microalgae using microwaves and water alone (Figure 1). Native spray-dried microalgae (also referred to as “standard” or the “standard method”) was subjected to microwave hydrothermal processing at a range of different temperatures (160–220 °C). The resultant hydrolysate and defibrillated celluloses were characterized using various techniques including: IR, TGA, XRD, ^13^C CPMAS NMR, TEM, WHC, and HPLC. The properties of these celluloses were compared with those produced from microalgal biomass that had undergone scCO_2_ extraction (Figure 1) because the latter is a widely used technique for isolating lipids, generating significant quantities of spent residues, which are often discarded. The valorisation of microalgae to defibrillated celluloses using hydrothermal microwave processing is new and significantly contributes to the literature on microalgal biorefineries.

## 2. Results and Discussion

### 2.1. Microfibrillated Cellulose (MFC) Yield and Carbohydrate Analysis

**Figure 2:**
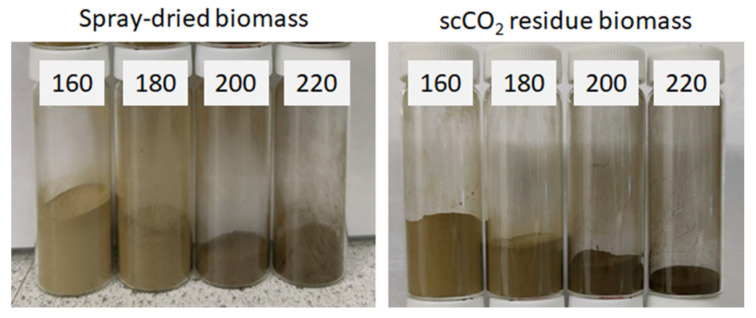
Microfibrillated cellulose (MFC) obtained via microwave treatment at temperatures of 160, 180, 200 and 220 °C for a total of 30 min (50:50 ramp:hold). Obtained from: Left—standard method using spray-dried microalgal biomass. Right: supercritical treated method using residual microalgal biomass post scCO_2_ extraction. NB. ‘Standard’ refers to without any scCO_2_ pretreatment.

MFC from untreated spray dried microalgal biomass (standard method) and scCO_2_ treated biomass were successfully generated during microwave processing at various temperatures (160–220 °C). As shown in Figure 2, an increased brown coloration was observed with increasing temperature due to degradation and caramelization of carbohydrates and their subsequent reaction with residual proteins (Maillard reaction) [18,19].

**Figure 3:**
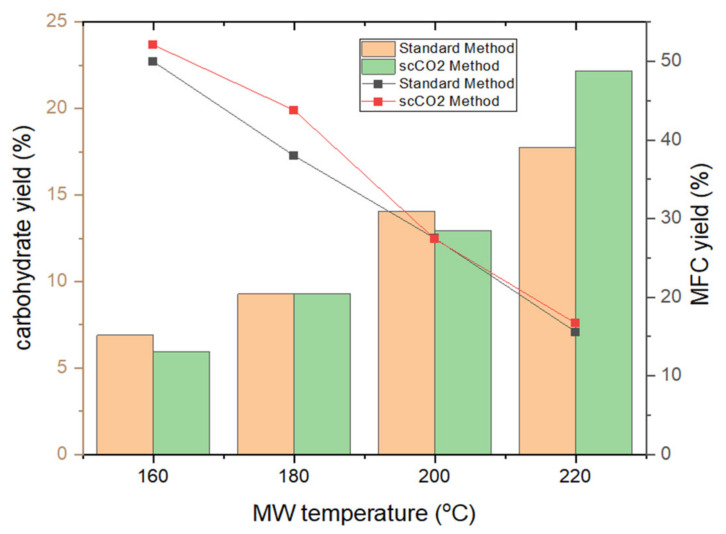
MFC yield (line chart) and carbohydrate yield (bar chart) of standard and supercritical treated microalgal biomass at different microwave temperatures. NB. ‘Standard’ refers to without any scCO_2_ pretreatment.

Figure 3 depicts the trends in MFC and carbohydrate yields following tangential ultrafiltration. The yield of MFC decreases by approximately 1 g per every 20 K increase in temperature reflecting the effect of microwave-induced degradation and removal of microalgal cell components such as lipids, pigments, hemicellulose and proteins [20,21,22]. Both the standard method and the scCO_2_ method result in similar MFC yields within a margin of fewer than 5% of each other, suggesting a limited effect of the supercritical treatment on MFC yield. Analogous with the degradation and defibrillation of cellulose, the carbohydrate yield similarly increases, as expected, in linear fashion, from 6% to a maximum of 22% [13,14,16].

**Figure 4:**
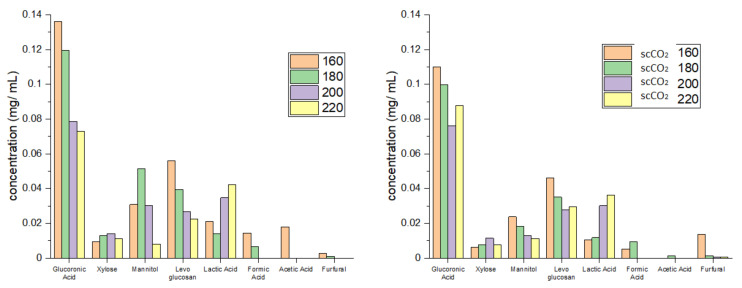
Carbohydrate split obtained from HPLC for standard method (**left**) and supercritical method (**right**). The numbers 160, 180, 200 and 220 refer to the microwave processing temperature (°C). NB. ‘Standard’ refers to without any scCO_2_ pretreatment.

Figure 4 shows the individual carbohydrate split obtained from HPLC analysis. High levels of glucuronic acid, an integral building block of the algal cell wall, which decreased with increasing temperature, were detected in hydrolysates from both standard and scCO_2_ treated algal biomass [23,24,25]. In contrast to glucuronic acid, the concentration of mannitol, levoglucosan and xylose was much lower but, nevertheless, increased with increasing temperature, peaking at 160 °C (standard method, levoglucosan), 180 °C (standard method, mannitol) and 200 °C (standard method, xylose). The formation of these sugars along with formic acid, furfural and acetic acid is consistent with the high temperature hydrolysis of cellulose and hemicellulose [13,14,16]. Furthermore, the concentration of lactic acid increased with increasing microwave temperature: again, this is consistent with depolymerization of carbohydrates [13,14,16].

### 2.2. Thermogravimetric Analysis

**Figure 5:**
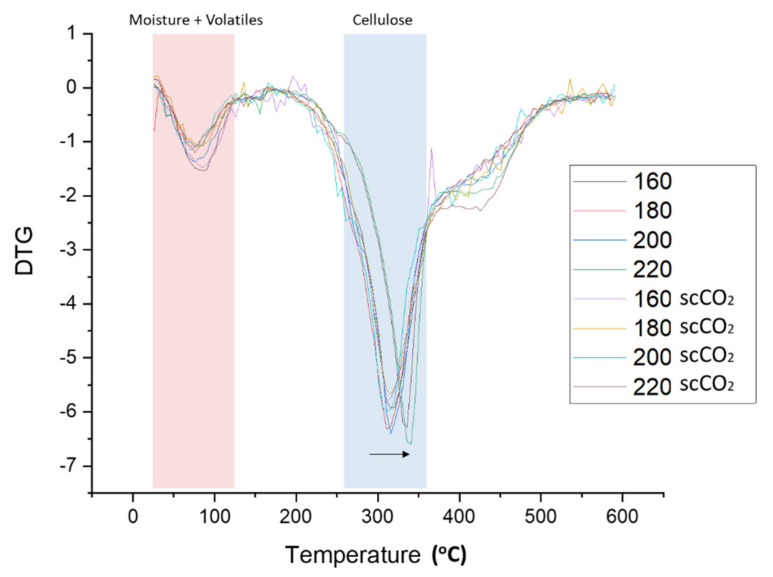
DTG thermograms of standard and scCO_2_ treated MFC. NB. ‘Standard’ refers to without any scCO_2_ pretreatment.

The rate of material weight change upon heating (DTG) is plotted against temperature for both the standard and scCO_2_-treated MFC are shown in Figure 5. Two major decomposition events were noted, namely: (i) loss of volatiles and moisture accounting for 4–8% of mass between 50–125 °C, and; (ii) cellulose decomposition accounting for 55–65% of mass loss between 280–390 °C. The temperature at which the rate of maximum decomposition, Td, occurs for cellulose remains relatively constant at approximately 315 °C for processing temperatures below 200 °C. However, at 220 °C the Td increased by approximately 30 °C to 345 °C (see Figure 5, black arrow). The heat-treatment induced restructuring of cellulose towards more crystalline structures as evidenced by XRD analysis and subsequent determination of the crystallinity index, as discussed next.

### 2.3. X-ray Powder Diffraction (XRD) Analysis and Crystallinity Index (CrI)

**Figure 6:**
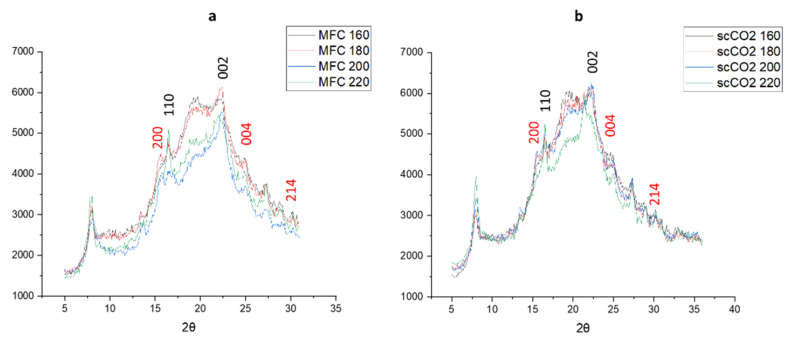
X-ray diffractograms of (**a**) standard method MFC and (**b**) scCO_2_ method MFC. Black numbers indicate cellulose planes, red numbers indicate CaC_2_O_4_ planes. NB. where ‘standard’ refers to without any scCO_2_ pretreatment.

The XRD patterns of materials produced from both the standard method and the scCO_2_ method are shown in Figure 6. The diffraction patterns arising from crystalline cellulose are marked in black numbers arising at 2θ = 16.5° and 22.5° [26,27,28]. With the higher microwave temperatures the intensity of the peak at 2θ = 16.5° increases, indicating a higher crystallinity, which is confirmed by the crystallinity index derived from the XRD patterns, as shown in Figure 6. Interestingly, the diffractograms for the standard method at 200 °C and 220 °C and those for the scCO_2_ method at 220 °C follow a slightly different pattern compared to their lower temperature counterparts, the latter showing more amorphous character and consistent with the thermogravimetric analysis discussed earlier in Figure 5.

The additional peaks that can be seen at 2θ = 15.1°, 24.4°, and 30° might indicate the presence of insoluble calcium salts, most notably calcium oxalate (CaC_2_O_4_) which can be present in microalgal cell structures, especially in vacuoles and the cell wall [14,29,30]. There does not seem to be any noticeable change in intensity for these calcium salt peaks, suggesting they are a constant component of microalgal MFC regardless of the temperature of the microwave treatment. 

**Figure 7:**
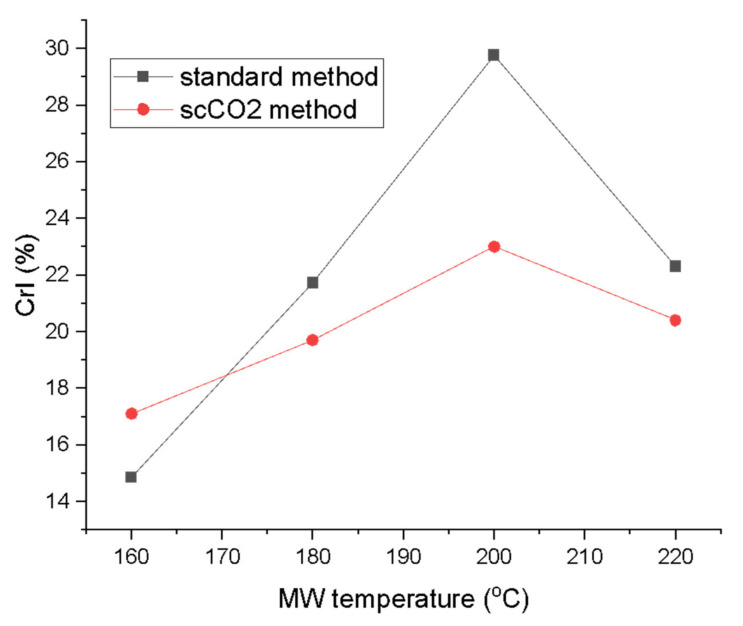
Crystallinity index (CrI) of MFC from standard and scCO_2_ methods at different MW temperatures calculated from XRD traces in Figure 6 via Segal’s method. NB. ‘Standard’ refers to without any scCO_2_ pretreatment.

The crystallinity index (CrI; Figure 7) derived from the XRD traces according to the Segal method revealed a steady increase in the crystallinity, peaking at 200 °C for both methods (29.8% for standard method and 23.0% for the scCO_2_ method). The CrI then drops for the highest microwave processing temperature to levels similar for materials processed at 180 °C [26,31,32]. Large differences in the CrI were also noted between the two processing methods. The standard method consistently yielded higher crystallinity from 180 °C onwards. The biggest difference in CrI at the same temperature for both methods was observed at 200 °C with ∆ = 6.8%. The crystallinity increased with temperature due to the gradual removal of amorphous impurities from the algal biomass such as starch, hemicellulose and amorphous cellulose from the cellulose matrix. The CrI values seem to be comparable to previous studies on pea, orange and ginger waste which, unlike microalgae, also contain lignin [13,14,16]. The drop in the CrI at the 220 °C can be explained by temperature-induced cellulose decomposition and restructuring, therefore, decreasing crystallinity [33,34].

### 2.4. ^13^C CPMAS Solid State NMR and TEM Imaging

**Figure 8:**
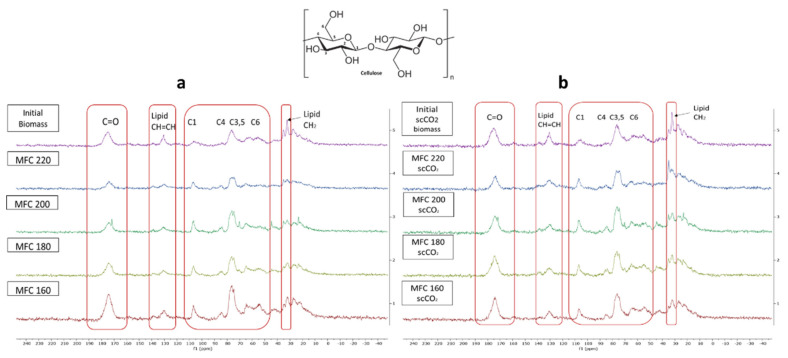
Solid state ^13^C CPMAS NMR spectra of (**a**) standard method MFC and (**b**) scCO_2_ method MFC. NB. ‘Standard’ refers to without any scCO_2_ pretreatment.

The stacked ^13^C CPMAS spectra for both methods are shown in Figure 8. The signal appearing at 175 ppm corresponds to the carbonyl carbon of carbonyl and carboxylic acid groups characteristic of hemicelluloses, pectins and, possibly, some polyunsaturated fatty acids (PUFAs) found in microalgal cell walls. The signals at around 130 ppm possibly indicate the presence of double bonded carbons which may correspond to PUFAs, which make up a large proportion of microalgal cell mass [35,36]. The intensity of these signals decrease with increasing microwave temperature suggesting the breakdown/removal of these possible lipids in the final MFC. Furthermore, the presence of a relatively strong signal at 32 ppm may correspond to unsaturated methylene carbon, which also consistently decreases in intensity with increasing microwave temperature.

Characteristic signals for cellulose carbons in the region between 120–60 ppm were observed and are assigned in the spectrum according to their corresponding position in the cellulose chain (C2–C6) [35,36]. Definite assignment and changes in the amorphous/crystalline structure are harder to observe due to the broad signals found in this region arising from residual amorphous regions which give rise to broader signals compared to sharper signals from crystalline cellulose [37,38]. However, similarly to previous findings, increasing microwave temperature results in an increased crystallinity, as evidenced by the presence of peaks at 65 ppm, which gain sharpness. The characteristic amorphous signals at 84 ppm and 62 ppm decrease slightly which mirrors the changes in the CrI displayed earlier in Figure 7. Moreover, the sharpness and resolution of the double peak at 77–74 ppm increases with increasing temperature up to 200 °C and, thereafter, becomes less defined at 220 °C. This correlates well changes in the CrI reported earlier, which increase to a maximum (200 °C) and then fall. 

**Figure 9:**
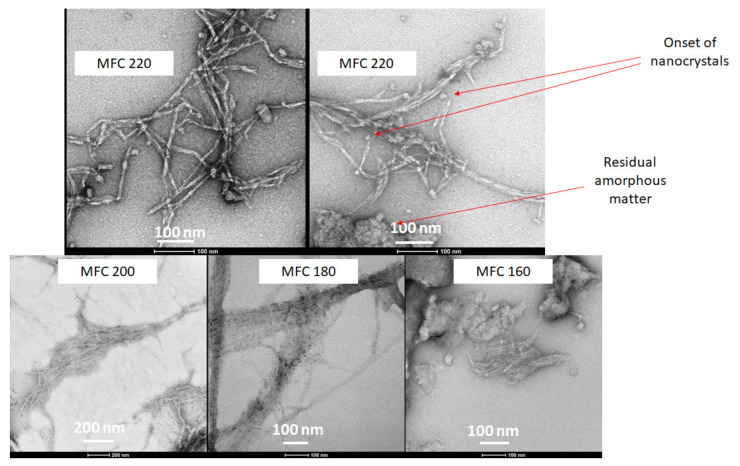
TEM images of MFC samples at different temperatures as labelled. MFCXXX corresponds to microfibrillated cellulose processed at XXX °C.

TEM gave a clear indication of the defibrillation of cellulose to afford fibres and potential crystals via the measurement of their aspect ratios. The width of the cellulose fibrils decreased from around 20–25 nm for the 160 and 180 °C samples to 7–8 nm for 200 °C, reaching a minimum width of 6 nm width at 220 °C. Furthermore, the very linear strand arrangement of the cellulose fibres, which can be seen very well in the 180 °C sample, is noticeably broken at the highest microwave temperature (220 °C). At the highest temperature (220 °C), fraying of the fibres was noticed to reveal the onset of nanocrystals (Figure 9). The TEM images correlate well with the CrI discussed earlier. The 180°C sample is highly ordered whilst the 220 °C sample is highly disordered.

A mechanistic interpretation of the defibrillation of microalgae is synonymous with the Hy-Mass (Hydrothermal Microwave Assisted Selective Scissoring) Effect reported by de Melo et al. for defibrillation of citrus fibres [13]. At temperatures below 180 °C significant leaching and breakdown of hemicellulosic and pectinaceous matter is noted, which predominantly leads to longitudinal scissoring of the cellulosic bundles. Above 180 °C, it is well known that microwave hydrolysis induces leaching and in situ acid hydrolysis of amorphous cellulose, which results in transverse scissoring of cellulose, ultimately leading to nanocrystals. 

### 2.5. Hydrogel Formation Capabilities and Water Holding Capacity (WHC)

**Figure 10:**
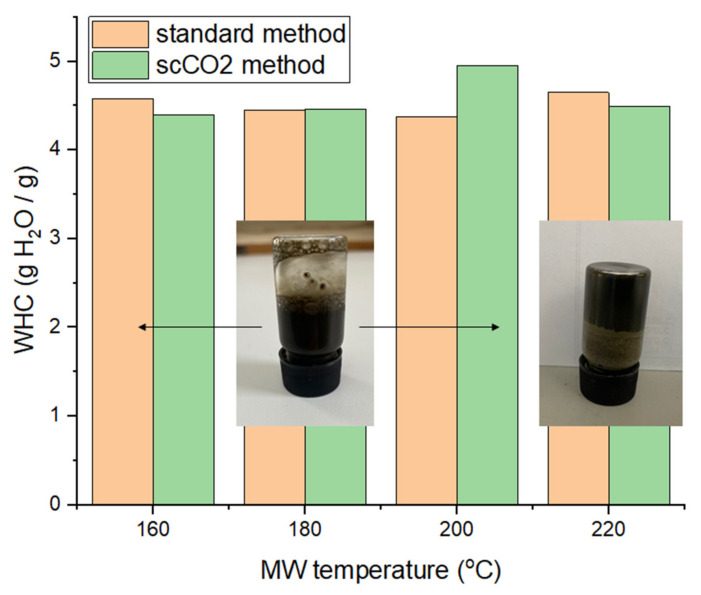
Water holding capacities (WHC) of MFC of both standard and scCO_2_ methods. NB. ‘Standard’ refers to without any scCO_2_ pretreatment.

The water holding capacity (WHC) of the different MFC samples are summarized in Figure 10. The WHC fluctuates around 4.5 g H_2_O/g sample, without any perceivable trend or difference with respect to the method used, i.e., ‘standard’ or scCO_2_ treated. These values are lower than for lignocellulosic or pectinaceous biomass derived MFCs, as shown by previous work, suggesting that the cellulosic framework generated from microalgal biomass is not able to hold as much water and may be more hydrophobic [13,14,16].

The ability to form hydrogels was conducted at various concentrations of MFC in deionized water (0.5%, 0.75%, 1%, 1.5%, 2% and 3%). Only the samples processed at 220 °C afforded pseudo-stable hydrogels at 3 wt% in water (see inset Figure 10). However, the gels only persist for approximately 10 s at the top of an inverted vial before sliding down. After a few hours of standing, the gel properties subside and the sample needs to be re-homogenized in order to regain the gelling properties. Nevertheless, the resultant materials have the ability to hold water and form hydrogels, thus opening new opportunities in many industrial sectors, such as food, pharmaceuticals and cosmetics, as coatings, films and barrier materials [14].

## 3. Materials and Methods

Microalgae was obtained from AlgaeCytes, Kent, England, who provided omega-3 enriched biomass from their proprietary microalgal *Eustigmatophyceae* strain, ALG01. The ALG01 strain was up-scaled from Petri dish to 100 L using AlgaeCytes in-house proprietary upstream pyramid process and inoculated into the 1000 L Industrial Plankton seeding tank. Once the culture reached late exponential phase, it was transferred into AlgaeCytes pilot plant production module (VariconAqua 12,000 L Phyco-Flow^TM^). After reaching an appropriate density, it underwent semi-continuous harvesting to provide material for spray drying. On each harvesting day, 1000 L of algal culture was dewatered using an Alfa Laval Clara 20 model disc-stack centrifuge to produce an algal slurry of ~15% +/− 5% solids. The algal slurry was subsequently dried using a Bϋchi mini spray dryer B-290 to produce a dried algal powder of <1% moisture content.

The term ‘standard’ refers to spray dried algae that was used for directly for acid-free, TEMPO-free hydrothermal microwave processing, i.e., without any pretreatment. Otherwise, the algae was subjected to scCO_2_ extraction to produce residues, which were then treated with acid-free, TEMPO-free hydrothermal microwave processing.

Supercritical extraction biomass residues were produced following a 2 h scCO_2_ extraction (300 bar, 50 °C, 30 g min^−1^) of the as-received spray-dried algae (50 g) using a supercritical extractor SFE-500 (Thar Technologies). Upon completion, the system was depressurized at a rate of 0.3 bar s^−1^ and the biomass collected (99.7 ± 0.1%).

Hydrothermal microwave treatment was carried out on a Milestone Synthwave reactor (1500 W, 2.45 GHz). Microalgae (10 g) was mixed with deionized water (350 mL) at a ratio of 1:35 (*w*/*v*) in a PTFE vessel (900 mL), and microwaved at different temperatures (160, 180, 200 and 220 °C) for a total time of 30 min (15 min ramp time, 15 holding time). The resulting slurry was centrifuged for 20 min on a Thermofisher Megafuge 4R for 20 min at 3600 rpm at room temperature. The supernatant and pellet were separated and the former subjected to microfiltration (pore size: 10 μm) on a KrosFlo Research Iii Tangential Flow Filtration System using a mPES MidiKros filter module. The resulting fraction was analysed using HPLC in order to evaluate its carbohydrate content. HPLC was run on an Agilent 1260 reverse phase LC system equipped with a Hi PLEX H+ column (300 × 7.7 mm, 8 μm particle size) maintained at 60 °C eluting with 0.005 M H_2_SO_4_ as the mobile phase. A run time of 30 min, flow rate of 0.4 mL/min, and an injection volume of 5 µL was employed.

The pellet was washed sequentially with hot water (300 mL, 15 min, 80 °C), hot ethanol (2 × 300 mL, 15 min, 65 °C), cold ethanol (300 mL, 15 min, 20 °C) and acetone (300 mL, 15 min, 20 °C), followed by air drying at ambient temperatures for 24 h to afford the desired microfibrillated cellulose (MFC). The yield of MFC was calculated according to Equation (1).

% Yield (MFC) = (dry mass of MFC/mass of raw biomass) × 100(1)

Thereafter, the various MFC samples were analysed via: a. Thermogravimetric analysis (TGA) on a Netzsch STA 409 instrument. The sample (~50 mg) was heated from 20 to 625 °C at 10 K min^−1^ under flow of nitrogen (100 mL min^−1^); b. X-ray powder diffraction (XRD) was run on a Bruker AXS D8 Advance Diffractrometer, with the samples having been finely ground prior to analysis and loaded onto a 0.75 mm thick sample holder. The samples were run using a locked-coupled scan type, with a scan speed of 0.1 s per step, voltage of 40 kV, and current of 40 mA; c. ^13^C cross polarization magic angle spinning (CPMAS) spectroscopy was performed on a Bruker JEOL 400S spectrometer with a ^13^C frequency of 10 kHz, a spin rate of 10,000 Hz, recycle delays of 5 s and a total number of 512 scans; d. Transmission electron spectroscopy (TEM) was run on a TEM Tecnai 12 BioTWIN instrument with a SIS Megaview 3 camera at a 76-acceleration voltage of 120 kV. A 2% mass ratio of the finely ground samples were dispersed in water and ultrasonicated in an ultrasound bath at 1500 W for 20 min to improve the image clarity. The water holding capacity (WHC) was determined by dispersing the appropriate dry MFC (2 g) water (38 mL) in a weighted centrifuge tube and shaking for 10 min. The resultant mixture was centrifuged (30 min at 3000 rpm), and supernatant was carefully removed from the wet pellet. The weight of wet pellet was determined and the WHC was calculated according to Equation (2).

WHC (g H_2_O/g sample) = (mass of wet sample + mass of tube and dry sample) mass of dried sample(2)

## 4. Conclusions

The valorisation of microalgae, often for their lipids, proteins and specialty bioactives, is well explored in the literature. However, there is minimal research focused on valorisation of the spent, cellulosic, residues. Our work has shown that these residues can be defibrillated using acid-free and TEMPO-free microwave processing to produce materials similar to micro- and nano-fibrillated cellulose, irrespective of the initial method, i.e., ‘standard’ or supercritical treated. The resultant materials have the ability to hold water and form hydrogels thus opening new opportunities in many industrial sectors such as food, pharmaceutical and cosmetics. However, this opportunity holds true only in an integrated biorefinery where initial capex costs for purchase of scCO_2_ extractors and hydrothermal microwave processors is factored into business calculations. The integrated biorefinery should already be using these technologies to valorise high value compounds and not just for making defibrillated celluloses. Furthermore, the cellulosic content in microalgae is limited compared to say, for example, waste paper. Thus, significantly higher volumes of spent microalgal residues will be needed compared to the same unit of waste paper to afford the same quantity of defibrillated cellulose. Nevertheless, this work significantly adds to new knowledge in the development of zero waste microalgal biorefineries.

## Data Availability

The data presented in this study is available to use. There is no additional supplementary information.

## Sample Availability

Samples of the defibrillated cellulose are available from the authors upon request.
